# Supplementary material for: The molecular basis for SARS-CoV-2 binding to dog ACE2
Source: Nat Commun. 2021 Jul 7;12:4195. doi: 10.1038/s41467-021-24326-y (PMC8263772; doi:10.1038/s41467-021-24326-y)
Supplement: Supplementary file 1 — Supplementary Information [file 41467_2021_24326_MOESM1_ESM.pdf]

Supplementary Information

Supplementary Figure 1

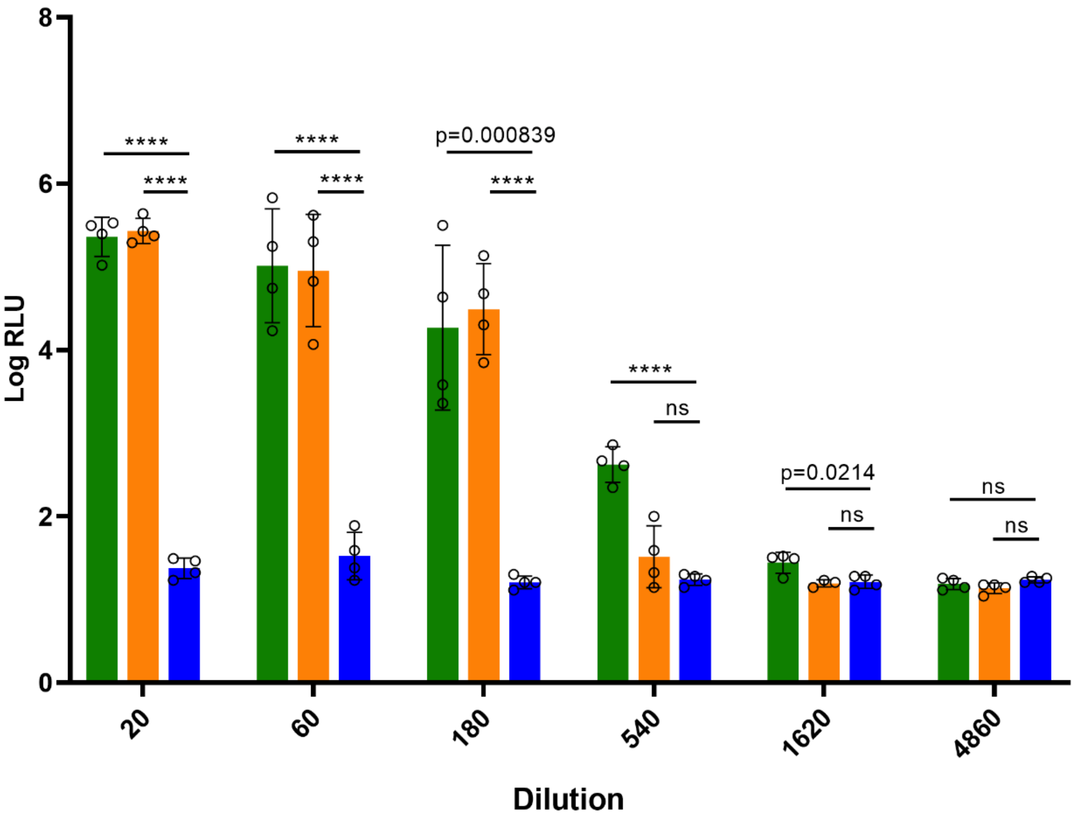

Supplementary Figure 1. Infection of dACE2- or hACE2-expressing HeLa cells with the SARS-CoV-2 S protein-bearing pseudovirus. Data are presented as mean values ± SD of quadruplicate cell samples. ****, $p < 0.0001$; ns means no significant differences; two-sided Student's *t*-test.

Supplementary Figure 2

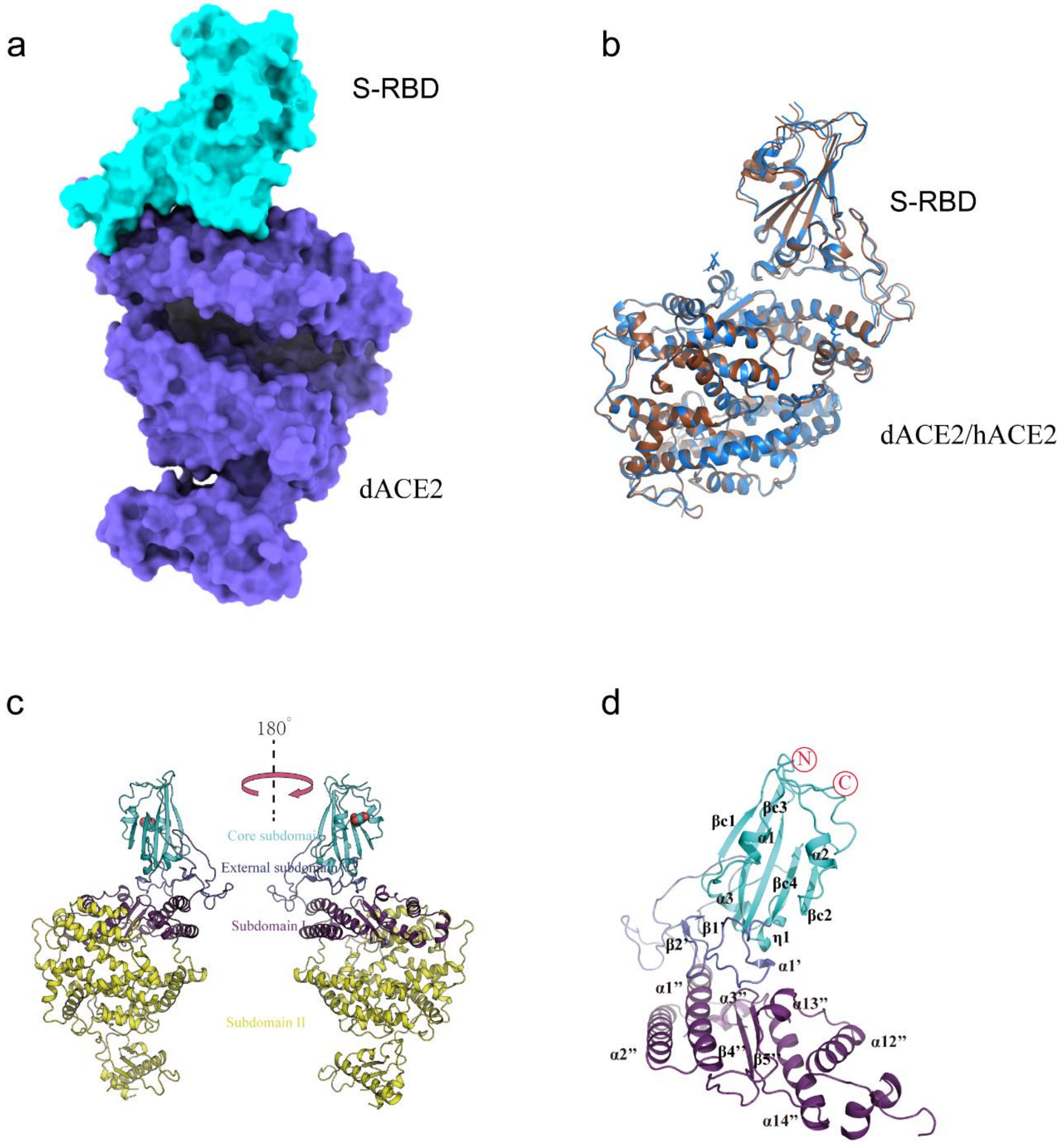

Supplementary Figure 2. The crystal structure of the RBD/dACE2 complex. (a) The overall crystal structure of the RBD/dACE2 complex. RBD is labeled in cyan and dACE2 in blue. (b) Alignment of the RBD/dACE2 complex (brown) to the RBD/hACE2 complex (marine, PDB ID: 6LZG). (c) The subdomains of RBD and dACE2 at two side viewpoints of 180° rotation. The RBD core subdomain, RBD external subdomain, dACE2 subdomain I and dACE2 subdomain II are colored with cyan, slate, purple, and yellow, respectively. (d) The secondary structural elements in

the RBD and dACE2 subdomain I in the complex. The subdomains are colored the same as in (c).

Supplementary Figure 3

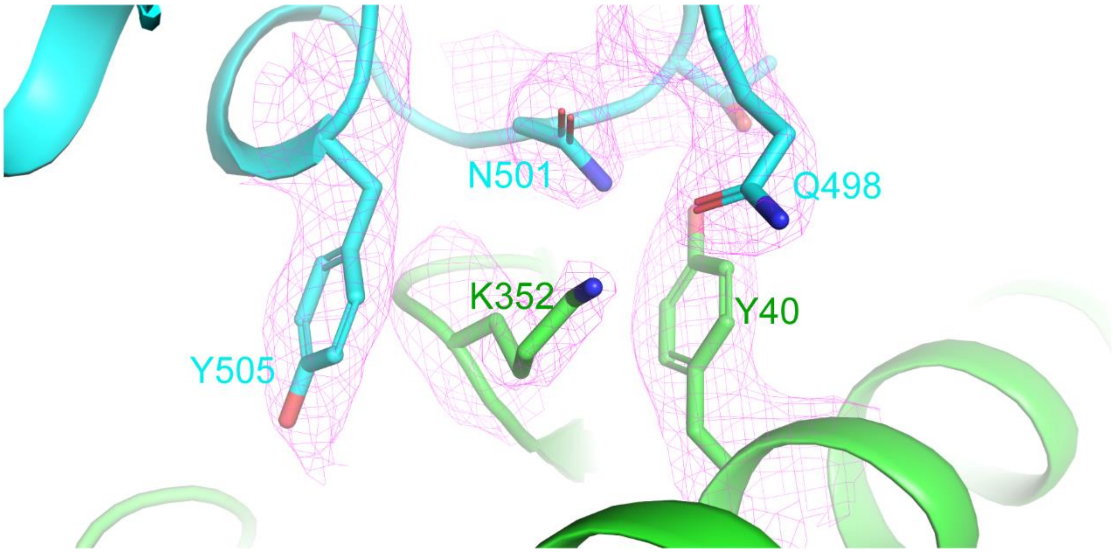

Supplementary Figure 3. The local 2Fo-Fc electronic density map at 1.0 σ for the binding interface in the RBD/ dACE2 complex. The electronic density is represented as pink mesh. RBD is shown as cyan and dACE2 is shown as green.

Supplementary Figure 4

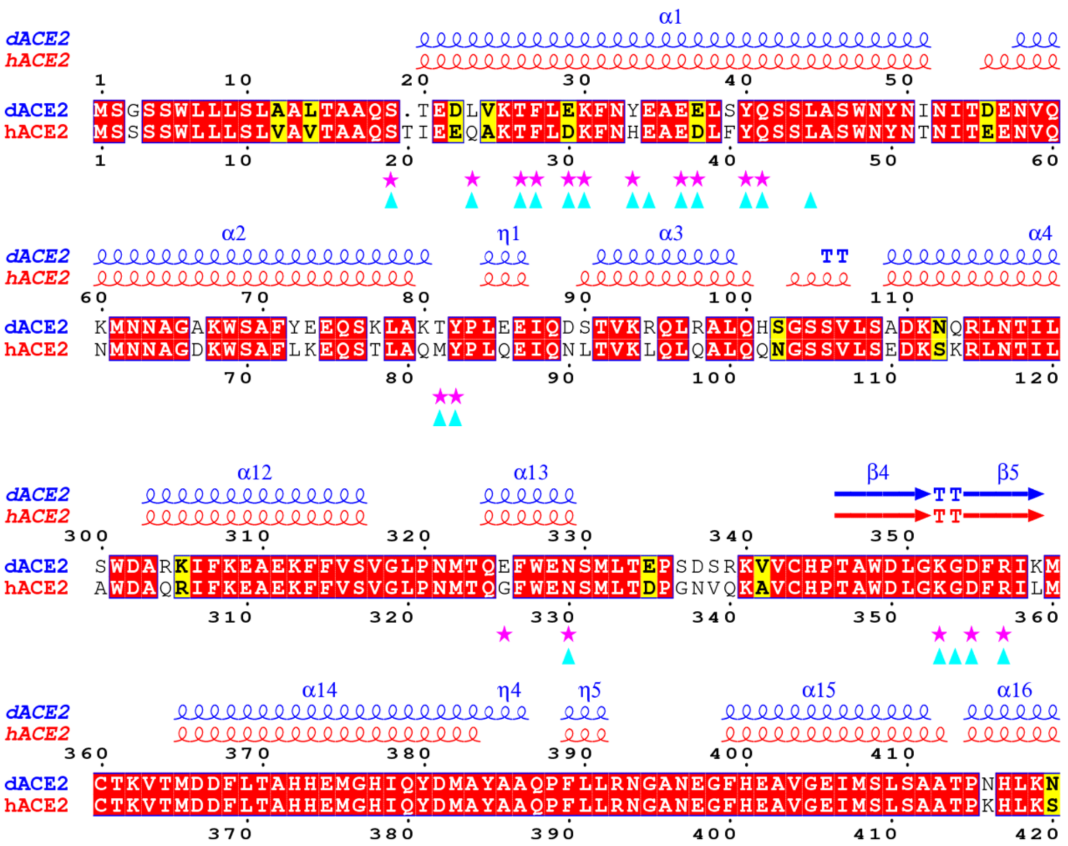

Supplementary Figure 4. Sequence alignment of dACE2 and hACE2. The dACE2 residues that contact with RBD in the RBD/ dACE2 are shown as hot pink stars. The hACE2 residues that make contact with RBD in the RBD/ hACE2 are shown as cyan triangles. The sequence alignment was performed with MEGAX [1] and visualized with ESPript [2]. The columns with residues in white and the background in red indicate a strict identity, and the columns with residues in black and the background in yellow indicate a similarity score over 0.7, considering the physio-chemical property.

Supplementary Figure 5

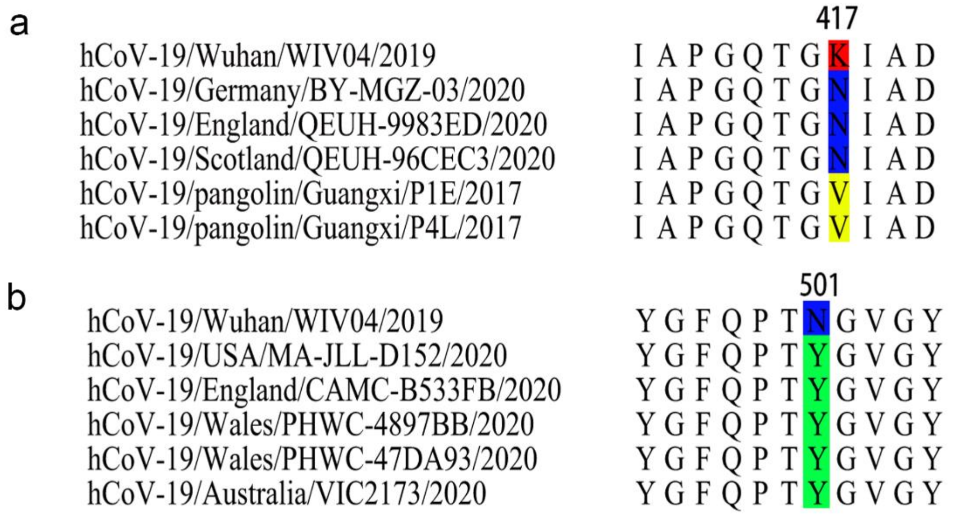

Supplementary Figure 5. Alignment of parts of the RBD amino acid sequences from different strains containing the relevant mutations tested in the present study. (a) Alignment of SARS-CoV-2 S protein containing the K417N and the K417V mutations with the reference sequence (hCoV-19/Wuhan/WIV04/2019). (b) Alignment of SARS-CoV-2 S protein contain the N501Y mutation with the reference sequence **(**hCoV-19/Wuhan/WIV04/2019). The sequence alignment was prepared with MEGAX [1]. The residue positions 417 (a) and 501 (b) are highlighted in different colors.

Supplementary Figure 6

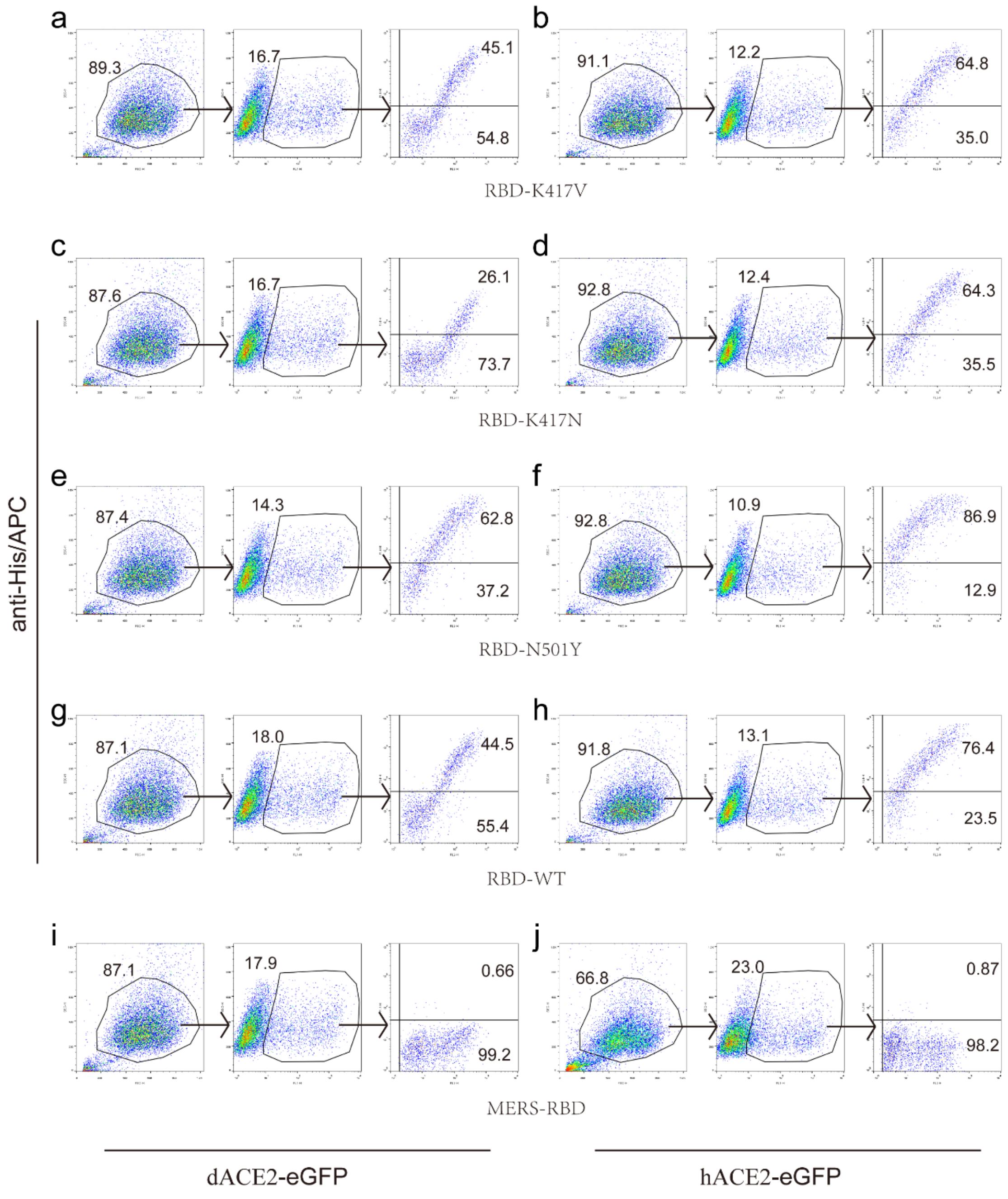

Supplementary Figure 6. The gating strategy for flow cytometry analysis of SARS-CoV-2 RBD interface residue mutants, wt RBD, or MERS-CoV RBD binding to BHK21 cells expressing dACE2 or hACE2. The cells expressing dACE-eGFP (a, c, e, g, i) or hACE-eGFP (b, d, f, h, j) were incubated with His-tagged RBD K417V mutant (a and b), RBD K417N mutant (c and d), RBD N501Y (e and f), wt RBD (g and h) or MERS-CoV RBD (I and j), respectively. The cells were the strained with the anti-

His/APC antibody and subjected to flow cytometry analysis. The cells populated based on the forward scatter (FSC) and the side scatter (SSC) signals were first gated with the eGFP fluorescent densities. The $eGFP^+$ cell population were further divided into the APC^+ population (cells binding to RBD or its mutants) and APC^- population (cells not binding to RBD or its mutants). The percentages of the APC^+ $eGFP^+$ cells and the APC^- $eGFP^+$ cells in the $eGFP^+$ cells are indicated.

Supplementary Table 1. Data collection and refinement statistics

	SARS2-CoV-2 RBD/dACE2
**Data collection**	
Space group	I422
Cell dimensions	
a, *b*, *c* (Å)	168.26, 168.26, 211.20
α, β, γ (°)	90.00, 90.00, 90.00
Resolution (Å)	50.00-3.00 (3.11-3.00)
Unique reflections	30355 (2989)
Completeness (%)	98.5 (99.5)
R_{merge}	0.18.4 (0.599)
$I/\sigma I$	8.8 (2.6)
$CC_{1/2}$ (%)	0.980 (0.819)
Redundancy	6.3 (6.2)
**Refinement**	
Resolution (Å)	48.26-3.00
No. reflections	29668
R_{work} / R_{free}	0.2272/0.2479
No. atoms	
Protein	7244
Ligand/ion	1
Water	0
B-factors	
Protein	52.6
Ligand/ion	76.8
Water	
R.M.S. deviations	
Bond lengths (Å)	0.002
Bond angles (°)	0.531
Ramchandran Statistics (%)	
Favored	96.49
Allowed	3.51
Disallowed	0.00

Values in parentheses are for the highest resolution shell.

Supplementary Table 2. Comparison of residue contacts of SARS-CoV-2 RBD binding to dACE2 and hACE2

SARS-CoV-2 RBD	dACE2	hACE2
R403 (1/0)	Y33(1)	-
K417 (2,1/3,1)	E29 (1,1)	D30 (3,2)
G446 (1,1/1,1)	Q41 (0,1)	Q42 (1)
Y449 (6,2/8,2)	E37 (4,1), Q41 (0,1)	D38 (5,1), Q42 (1,1)
Y453 (6/3)	Y33 (6)	H34 (3)
L455 (3/4)	E29(1), Y33 (2)	H34 (4)
F456 (4/7)	T26 (3), E29 (1)	T27 (5), D30 (1), K31 (1)
A475 (3/5,1)	L23(2), T26(1)	S19 (3,1), Q24 (1), T27 (1)
G476 (0/2)		S19 (2)
F486 (10/11)	T81(3), T82 (7)	M82 (4), Y83 (7)
N487 (4,1/11,2)	L23 (1), Y82 (3,1)	Q24 (7,1), Y83 (4,1)
Y489 (7/7,1)	T26 (1), F27 (3), K30 (2), Y82 (1)	T27 (2), F28 (4), Y83 (0,1)
F490 (0/1)		K31 (0,1)
Q493 (0/8,1)		H34 (3), E35 (4,1)
G496 (6,1/2,1)	E37 (2), K352 (4,1)	D38 (1), K353 (1,1)
Q498 (10,2/19,1)	E37 (2,1), Y40(3), Q41 (2, 1), K352(3,1)	D38(1), Y41(7), Q42(9,1), L45(2)
T500 (17,2/17,1)	Y40 (6,1), N329(4), D354 (6,1), R356 (1)	Y41 (5,1), N330 (3), D355 (5), R357 (3)
N501 (11,1/10,2)	Y40 (5,1), E325(1), K352(5)	Y41 (5,1), K353 (4,1)
G502 (7,1/8,1)	K352 (2,1), G353(5)	K353 (3,1), G354 (5)
Y505 (27,2/20,1)	E36(7,1), K352 (18), R392(1,1)	E37 (2,1), K353 (15), G354 (2), R392(1)
Q506(1,1/0)	E325(1,1)	
Total	127, 13	145, 16

The numbers in parentheses of SARS-CoV-2 RBD residues represent the number of vdw and H-bond/salt bridges contacts between the indicated residue with dACE2 (the former) and hACE2 (the latter). The numbers in parentheses of ACE2 residues represent the numbers of vdw contacts the indicated residues conferred. The numbers with underlines suggest numbers of potential H-bonds or salt bridges between the pairs of residues. Vdw contacts were analyzed at a cutoff of 4 Å. H-bonds/salt bridges were analysis with PDBePISA (https://www.ebi.ac.uk/pdbe/pisa/)

Supplementary Table 3. Hydrogen bonds and salt bridges at the binding interfaces of in the dACE2/SARS-CoV-2 RBD and the hACE2/ SARS-CoV-2 RBD complexes (*)

		dACE2/SARS-CoV-2 RBD complex							hACE2/ SARS-CoV-2 RBD complex				
		Chain B		Distance	Chain A						Distance		
	No.	Residue	Atom		Residue	Atom		No.	Residue	Atom		Residue	Atom
	1	K417	NZ	3.25	E29	OE1		1	K417	NZ	2.86	D30	OD1
	2	Y449	OH	2.66	E37	OE2		2	Y449	OH	3.09	Q42	OE1
	3	N487	ND2	3.16	Y82	OH		3	Y449	OH	2.83	D38	OD1
	4	Q498	NE2	2.92	E37	OE2		4	N487	ND2	2.82	Y83	OH
	5	T500	OG1	2.43	Y40	OH		5	Y489	OH	3.54	Y83	OH
	6	N501	N	3.73	Y40	OH		6	Q493	NE2	3.39	E35	OE1
Hydrogen bond	7	G502	N	2.92	K352	O		7	Q498	NE2	2.57	Q42	OE1
	8	Y505	OH	2.52	E36	OE2		8	T500	OG1	2.73	Y41	OH
	9	Q506	NE2	3.59	E325	OE2		9	N501	N	3.62	Y41	OH
	10	G446	O	3.61	Q41	NE2		10	G502	N	2.79	K353	O
	11	Y449	OH	3.36	Q41	NE2		11	Y505	OH	3.66	E37	OE1
	12	G496	O	2.97	K352	NZ		12	A475	O	2.81	S19	OG
	13	Y505	OH	3.6	R392	NH2		13	N487	OD1	2.87	Q24	NE2
								14	F490	O	3.82	K31	NZ
								15	G496	O	3.22	K353	NZ
Salt bridge	1	K417	NZ	3.25	E29	OE1		1	K417	NZ	3.76	D30	OD2
								2	K417	NZ	2.86	D30	OD1

(*) H-bonds/salt bridges were analysis with PDBePISA (https://www.ebi.ac.uk/pdbe/pisa/)

Supplementary Table 4. Codon optimized gene sequence for proteins expression

1. dACE2, tagged with 6xHis, cloned into pET21a with NdeI and Xhol
CATATGCAGTCAACAGAAGATCTTGTTAAGACGTTCCTTGAGAAGTTCAATTATGAAGCTGAAGAACTTTCATAT<br>CAGTCATCACTTGCTTCATGGAACTATAACATTAACATTACAGATGAGAATGTCCAGAAGATGAATAACGCTGGC<br>GCTAAATGGTCAGCTTTCTACGAGGAACAGTCAAAGCTAGCAAAGACTTACCCTCTTGAAGAAATTCAGGATTC<br>AACAGTTAAACGCCAGCTTCGCGCTCTTCAGCATTCAGGCTCATCAGTTCTTTCAGCTGATAAGAATCAACGCCT<br>TAACACAATTCTTAACTCAATGTCAACAATTTATTCAACAGGCAAAGCTTGTAACCCTTCAAACCCTCAGGAATG<br>TCTTCTTCTTGAACCTGGTCTGGACGACATAATGGAGAATAGTAAGGACTACAACGAACGCCTTTGGGCTTGGGA<br>AGGCTGGCGCTCAGAAGTTGGCAAACAGCTTCGCCCTCTTTATGAAGAATATGTTGCTCTTAAGAATGAGATGGC<br>TCGCGCTAACAACTATGAAGATTATGGCGATTATTGGCGCGGCGATTATGAAGAAGAATGGGAGAATGGATATAA<br>CTATTCACGCAACCAGCTTATTGATGATGTTGAACATACATTTACACAGATTATGCCTCTTTATCAGCATCTTCATG<br>CTTATGTTCGCACAAAGCTAATGGATACATATCCTTCATATATTTCACCTACAGGCTGTCTTCCTGCTCATCTTCTTG<br>GCGATATGTGGGGACGGTTCTGGACCAACCTTTATCCTCTTACAGTTCCTTTCGGTCAGAAGCCAAATATTGATGT<br>TACAAACGCTATGGTTAACCAGTCATGGGATGCTCGCAAGATCTTTAAGGAGGCTGAGAAGTTCTTCGTTTCGGT<br>TGGCCTTCCTAACATGACACAGGAATTCTGGGAGAACTCAATGCTTACAGAACCTTCAGATTCACGCAAAGTTGT<br>TTGTCATCCTACAGCTTGGGATCTTGGCAAAGGCGATTTCCGGATCAAGATGTGCACAAAGGTGACGATGGATGA<br>TTTCCTGACCGCTCATCATGAAATGGGCCATATTCAGTATGATATGGCTTATGCTGCTCAGCCTTTCCTCTTACGCA<br>ACGGCGCTAACGAAGGCTTTCATGAAGCTGTTGGCGAAATTATGTCACTTTCAGCTGCTACACCTAACCATCTTA<br>AGAATATAGGCCTTCTTCCTCCTTCATTCTTCGAGGACTCAGAAACAGAAATTAACTTTCTTCTTAAACAGGCTCT<br>TACAATTGTTGGCACACTTCCTTTCACGTACATGCTTGAGAAGTGGCGTTGGATGGTATTCAAGGGTGAGATCCC<br>TAAAGATCAGTGGATGAAGACTTGGTGGGAAATGAAACGCAACATTGTTGGCGTTGTTGAACCTGTTCCTCATG<br>ATGAAACATATTGTGATCCTGCTTCACTGTTCCACGTGGCTAACGATTATTCATTTATTCGCTATTATACACGCACA<br>ATTTATCAGTTTCAGTTTCAGGAAGCTCTTTGTCAGATTGCTAAACATGAAGGCCCTCTTCATAAATGTGATATTTC<br>AAACTCATCAGAAGCTGGCCAGAAACTTCTTGAAATGCTTAAACTTGGCAAATCAAAGCCGTGGACTTATGCTC<br>TTGAAATTGTTGTTGGCGCTAAGAATATGGATGTTCGCCCTCTTCTTAACTATTTCGAGCCGCTCTTCACTTGGCTT<br>AAAGAACAGAACCGCAACTCATTTGTTGGCTGGAACACAGATTGGTCACCTTATGCTGATCAGTCAATTAAAGTT<br>CGCATTTCACTTAAATCAGCTCTTGGCGAGAAGGCCTACGAATGGAACAACAACGAAATGTATCTCTTCAGATCT<br>TCAATTGCTTATGCTATGCGCCAGTATTTCTCCGAGGTTAAGAATCAAACAATTCCTTTCGTCGAGGATAACGTTT<br>GGGTTTCAGATCTTAAACCTCGCATTTCATTTAACTTCTTCGTCACCTCACCTGGCAACGTTTCAGATATTATTCCT<br>CGCACAGAAGTTGAAGAAGCTATTCGCATGTATCGCTCACGCATTAACGATGTATTCCGGCTAGACGACAATAGT<br>CTTGAATTTCTTGGCATTCAGCCTACACTTGGCCCTCCTTATGAACCTCCTGTTACACATCATCATCATCATCATTG<br>ACTCGAG

2. hACE2, fused with mFc, cloned into pCAGGS with NdeI and Xhol

CATATGTCAACAATTGAAGAACAGGCTAAGACCTTCCTTGATAAATTTAACCATGAAGCTGAAGATCTCTTCTACC
AATCATCACTTGCTTCATGGAACTATAACACAAACATTACAGAAGAGAATGTCCAGAACATGAACAACGCTGGC
GATAAATGGTCAGCTTTCTTGAAGGAACAGTCAACACTTGCTCAGATGTATCCTCTTCAGGAAATTCAGAACCTT
ACAGTTAAACTTCAGCTTCAGGCTCTTCAGCAGAATGGTTCTTCGGTGTTATCAGAAGATAAATCAAAGAGGCTC
AACACAATTCTTAACACAATGTCAACAATTTATTCAACAGGCAAAGTTTGTAACCCTGATAACCCTCAGGAATGT
CTTCTTCTTGAACCTGGCCTTAACGAAATTATGGCTAACTCACTTGATTATAACGAACGCCTTTGGGCTTGGGAAT
CATGGCGCTCAGAAGTTGGCAAACAGCTTCGCCCACTCTATGAGGAGTACGTTGTTCTTAAGAATGAGATGGCTC
GCGCTAACCATTATGAAGATTATGGCGATTATTGGCGCGGCGATTATGAAGTTAACGGCGTTGATGGCTATGATTAT
TCACGCGGCCAGCTTATTGAAGATGTTGAACATACATTTGAAGAAATTAAACCGCTCTACGAGCACTTGCATGCT
TATGTTCGCGCTAAACTTATGAACGCTTATCCTTCATATATTTCACCTATTGGCTGTCTTCCTGCTCATCTTCTTGGC
GATATGTGGGGTCGTTTTTGGACGAACCTTTATTCACTTACAGTTCCTTTCGGACAGAAGCCGAATATTGATGTTA
CAGATGCTATGGTTGATCAGGCTTGGGATGCTCAGCGCATATTCAAGGAGGCTGAGAAGTTCTTCGTTTCGGTTG
GCCTTCCTAACATGACACAGGGCTTCTGGGAGAACTCAATGCTTACAGATCCTGGCAACGTTCAGAAAGCTGTT
TGTCATCCTACAGCTTGGGATCTTGGCAAAGGCGATTTCAGGATCCTTATGTGTACAAAGGTAACTATGGATGATT
TCCTAACCGCACACCACGAGATGGGCCATATTCAGTATGATATGGCTTATGCTGCTCAGCCTTTCCTGTTACGCAA
CGGCGCTAACGAAGGCTTTCATGAAGCTGTTGGCGAAATTATGTCACTTTCAGCTGCTACACCTAAACATCTTAA
ATCAATTGGCCTTCTTTCACCTGATTTCCAAGAGGATAACGAAACAGAAATTAACTTTCTTCTTAAACAGGCTCTT
ACAATTGTTGGCACACTTCCTTTCACCTACATGCTTGAGAAGTGGCGGTGGATGGTATTCAAGGGTGAAATTCCT
AAAGATCAGTGGATGAAGAAGTGGTGGGAAATGAAACGCGAAATTGTTGGCGTTGTTGAACCTGTTCCTCATGA
TGAAACATATTGTGATCCTGCTTCATTGTTCCACGTATCAAACGATTATTCATTTATTCGCTATTATACACGCACACT
TTATCAGTTTCAGTTTCAGGAAGCTCTTTGTCAGGCTGCTAAACATGAAGGCCCTCTTCATAAATGTGATATTTCA
AACTCAACAGAAGCTGGCCAGAAACTGTTCAATATGCTTCGCCTTGGCAAATCAGAACCTTGGACACTTGCTCT
TGAGAATGTAGTTGGCGCTAAGAATATGAACGTTCGCCCTCTTCTTAACTATTTCGAGCCGTTATTCACGTGGCTT
AAAGATCAGAACAAGAATAGTTTCGTAGGGTGGTCAACAGATTGGTCACCTTATGCTGATCATCATCATCATCATC
ATTGACTCGAG

3. SARS-CoV-2 RBD wt, tagged with 6xHis, cloned into pCAGGS with EcoRI and Xhol

ATGTTTGTGTTTCTTGTGCTTCTTCCTCTTGTGTCATCACAATGCAGAGTGCAACCTACAGAATCAATCGTGAGAT
TTCCTAACATCACAAACCTTTGCCCTTTCGGCGAGGTGTTTAACGCAACAAGATTTGCATCAGTGTACGCATGGA
ACAGAAAGCGTATATCAAACTGCGTGGCAGATTACTCAGTGCTTTACAACTCAGCATCATTCAGTACGTTTAAAT
GCTACGGAGTGTCACCTACAAAGCTAAATGATCTTTGCTTTACAAACGTGTACGCAGATTCATTTGTGATCAGAG
GAGATGAAGTGAGACAAATCGCACCTGGACAAACAGGAAAGATTGCCGATTACAACTACAAACTTCCTGATGAT
TTCACCGGCTGCGTGATCGCATGGAACTCAAACAACCTTGATTCAAAGGTAGGTGGTAATTATAATTATTTGTATA
GGCTCTTTCGTAAGAGCAACTTAAAGCCATTTGAGCGAGATATCTCAACAGAAATCTACCAAGCAGGATCAACA
CCTTGCAACGGAGTGGAAGGATTTAACTGCTACTTTCCTCTTCAATCATACGGATTTCAACCTACAAACGGAGTG
GGATACCAACCTTACAGAGTGGTGGTGCTTTCATTTGAACTTCTTCACGCACCTGCAACAGTGTGCGGACCTAAG
AAGAGCACGAACCTTGTGAAGAATAAGTGCGTGAACTTTCACCACCACCACCACCACTGA

4. SARS-CoV-2 RBD wt, tagged with 6xHis, cloned into pFASTBAC^{TM}1 with EcoRI and Xhol

ATGTTTGTGTTTCTTGTGCTTCTTCCTCTTGTGTCATCACAATGCAGAGTGCAACCTACAGAATCAATCGTGAGAT
TTCCTAACATCACAAACCTTTGCCCTTTCGGCGAGGTGTTTAACGCAACAAGATTTGCATCAGTGTACGCATGGA
ACAGAAAGCGTATATCAAACTGCGTGGCAGATTACTCAGTGCTTTACAACTCAGCATCATTCAGTACGTTTAAAT
GCTACGGAGTGTCACCTACAAAGCTAAATGATCTTTGCTTTACAAACGTGTACGCAGATTCATTTGTGATCAGAG
GAGATGAAGTGAGACAAATCGCACCTGGACAAACAGGAAAGATTGCCGATTACAACTACAAACTTCCTGATGAT
TTCACCGGCTGCGTGATCGCATGGAACTCAAACAACCTTGATTCAAAGGTAGGTGGTAATTATAATTATTTGTATA
GGCTCTTTCGTAAGAGCAACTTAAAGCCATTTGAGCGAGATATCTCAACAGAAATCTACCAAGCAGGATCAACA
CCTTGCAACGGAGTGGAAGGATTTAACTGCTACTTTCCTCTTCAATCATACGGATTTCAACCTACAAACGGAGTG
GGATACCAACCTTACAGAGTGGTGGTGCTTTCATTTGAACTTCTTCACGCACCTGCAACAGTGTGCGGACCTAAG
AAGAGCACGAACCTTGTGAAGAATAAGTGCGTGAACTTTCACCACCACCACCACCACTGA

5. SARS-CoV-2 RBD K417V, tagged with 6xHis, cloned into pCAGGS with EcoRI and Xhol

GAATTCGCCACCATGTTTGTGTTTCTTGTGCTTCTTCCTCTTGTGTCATCACAATGCAGAGTGCAACCTACAGAAT
CAATCGTGAGATTTCCTAACATCACAAACCTTTGCCCTTTCGGCGAGGTGTTTAACGCAACAAGATTTGCATCAG
TGTACGCATGGAACAGAAAGCGTATATCAAACTGCGTGGCAGATTACTCAGTGCTTTACAACTCAGCATCATTCA
GTACGTTTAAATGCTACGGAGTGTCACCTACAAAGCTAAATGATCTTTGCTTTACAAACGTGTACGCAGATTCATT
TGTGATCAGAGGAGATGAAGTGAGACAAATCGCACCTGGACAAACAGGAGTGATTGCCGATTACAACTACAAA
CTTCCTGATGATTTCACCGGCTGCGTGATCGCATGGAACTCAAACAACCTTGATTCAAAGGTAGGTGGTAATTATA
ATTATTTGTATAGGCTCTTTCGTAAGAGCAACTTAAAGCCATTTGAGCGAGATATCTCAACAGAAATCTACCAAGC
AGGATCAACACCTTGCAACGGAGTGGAAGGATTTAACTGCTACTTTCCTCTTCAATCATACGGATTTCAACCTAC
AAACGGAGTGGGATACCAACCTTACAGAGTGGTGGTGCTTTCATTTGAACTTCTTCACGCACCTGCAACAGTGT
GCGGACCTAAGAAGAGCACGAACCTTGTGAAGAATAAGTGCGTGAACTTTCACCACCACCACCACCACTGACT
CGAG

6. SARS-CoV-2 RBD K417N, tagged with 6xHis, cloned into pCAGGS with EcoRI and Xhol

GAATTCGCCACCATGTTTGTGTTTCTTGTGCTTCTTCCTCTTGTGTCATCACAATGCAGAGTGCAACCTACAGAAT
CAATCGTGAGATTTCCTAACATCACAAACCTTTGCCCTTTCGGCGAGGTGTTTAACGCAACAAGATTTGCATCAG
TGTACGCATGGAACAGAAAGCGTATATCAAACTGCGTGGCAGATTACTCAGTGCTTTACAACTCAGCATCATTCA
GTACGTTTAAATGCTACGGAGTGTCACCTACAAAGCTAAATGATCTTTGCTTTACAAACGTGTACGCAGATTCATT
TGTGATCAGAGGAGATGAAGTGAGACAAATCGCACCTGGACAAACAGGAAACATTGCCGATTACAACTACAAA
CTTCCTGATGATTTCACCGGCTGCGTGATCGCATGGAACTCAAACAACCTTGATTCAAAGGTAGGTGGTAATTATA
ATTATTTGTATAGGCTCTTTCGTAAGAGCAACTTAAAGCCATTTGAGCGAGATATCTCAACAGAAATCTACCAAGC
AGGATCAACACCTTGCAACGGAGTGGAAGGATTTAACTGCTACTTTCCTCTTCAATCATACGGATTTCAACCTAC
AAACGGAGTGGGATACCAACCTTACAGAGTGGTGGTGCTTTCATTTGAACTTCTTCACGCACCTGCAACAGTGT
GCGGACCTAAGAAGAGCACGAACCTTGTGAAGAATAAGTGCGTGAACTTTCACCACCACCACCACCACTGACT
CGAG

7. SARS-CoV-2 RBD 501Y, tagged with 6xHis, cloned into pCAGGS with EcoRI and XhoI

GAATTCGCCACCATGTTTGTGTTTCTTGTGCTTCTTCCTCTTGTGTCATCACAATGCAGAGTGCAACCTACAGAAT
CAATCGTGAGATTTCCTAACATCACAAACCTTTGCCCTTTCGGCGAGGTGTTTAACGCAACAAGATTTGCATCAG
TGTACGCATGGAACAGAAAGCGTATATCAAACTGCGTGGCAGATTACTCAGTGCTTTACAACTCAGCATCATTCA
GTACGTTTAAATGCTACGGAGTGTCACCTACAAAGCTAAATGATCTTTGCTTTACAAACGTGTACGCAGATTCATT
TGTGATCAGAGGAGATGAAGTGAGACAAATCGCACCTGGACAAACAGGAAAGATTGCCGATTACAACTACAAA
CTTCCTGATGATTTCACCGGCTGCGTGATCGCATGGAACTCAAACAACCTTGATTCAAAGGTAGGTGGTAATTATA
ATTATTTGTATAGGCTCTTTCGTAAGAGCAACTTAAAGCCATTTGAGCGAGATATCTCAACAGAAATCTACCAAGC
AGGATCAACACCTTGCAACGGAGTGGAAGGATTTAACTGCTACTTTCCTCTTCAATCATACGGATTTCAACCTAC
ATACGGAGTGGGATACCAACCTTACAGAGTGGTGGTGCTTTCATTTGAACTTCTTCACGCACCTGCAACAGTGTG
CGGACCTAAGAAGAGCACGAACCTTGTGAAGAATAAGTGCGTGAACTTTCACCACCACCACCACCACTGACTC
GAG

8. MERS-CoV RBD, tagged with 6xHis, cloned into pCAGGS with EcoRI and XhoI

GAATTCGCAAAACCTTCTGGCTCAGTTGTGGAACAGGCTGAAGGTGTTGAATGTGATTTTTCACCTCTTCTGTCT
GGCACACCTCCTCAGGTTTATAATTTCAAGCGTTTGGTTTTTACCAATTGCAATTATAATCTTACCAAATTGCTTTC
ACTTTTTTCTGTGAATGATTTTACTTGTAGTCAAATATCTCCAGCAGCAATTGCTAGCAACTGTTATTCTTCACTGA
TTTTGGATTACTTTTCATACCCACTTAGTATGAAATCCGATCTCAGTGTTAGTTCTGCTGGTCCAATATCCCAGTTT
AATTATAAACAGTCCTTTTCTAATCCCACATGTTTGATTTTAGCGACTGTTCCTCATAACCTTACTACTATTACTAAG
CCTCTTAAGTACAGCTATATTAACAAGTGCTCTCGTCTTCTTTCTGATGATCGTACTGAAGTACCTCAGTTAGTGA
ACGCTAATCAATACTCACCCTGTGTATCCATTGTCCCATCCACTGTGTGGGAAGACGGTGATTATTATAGGAAACA
ACTATCTCCACTTGAAGGTGGTGGCTGGCTTGTTGCTAGTGGCTCAACTGTTGCCATGACTGAGCAATTACAGAT
GGGCTTTGGTATTACAGTTCAATATGGTACAGACACCAATAGTGTTTGCCCCAAGCTTGAATTTGCTAATGACACA
AAAATTGCCTCTCAATTAGGCAATTGCGTGGAATATTCCCACCACCACCACCACCACTGACTCGAG

Supplementary References

1. Kumar S, Stecher G, Li M, Knyaz C, Tamura K. MEGA X: molecular evolutionary genetics analysis across computing platforms. *Mol Biol Evol* **35**, 1547-1549. (2018).

2. Robert X, Gouet P. Deciphering key features in protein structures with the new ENDscript server. *Nucleic Acids Res* **42**, W320-324. (2014).
